# Sex-Specific Association of Uric Acid and Kidney Function Decline in Taiwan

**DOI:** 10.3390/jpm11050415

**Published:** 2021-05-15

**Authors:** Po-Ya Chang, Yu-Wei Chang, Yuh-Feng Lin, Hueng-Chuen Fan

**Affiliations:** 1 Department of Leisure Industry and Health Promotion, National Taipei University of Nursing and Health Sciences, Taipei 112, Taiwan; 2 Department of Business Management, National Taichung University of Science and Technology, Taichung City 404, Taiwan; nickychang@nutc.edu.tw; 3 Graduate Institute of Clinical Medicine, Taipei Medical University, Taipei 110, Taiwan; linyfmd@tmu.edu.tw; 4 Department of Pediatrics, Tungs’ Taichung Metroharbor Hospital, Taichung 435, Taiwan; t11578@ms.sltung.com.tw

**Keywords:** hyperuricemia, sex, renal progression, chronic kidney disease

## Abstract

An elevated serum urate concentration is associated with kidney damage. Men’s uric acid levels are usually higher than women’s. However, postmenopausal women have a higher risk of gout than men, and comorbidities are also higher than in men. This study examined the sex differences in the relationship between hyperuricemia and renal progression in early chronic kidney disease (CKD) and non-CKD, and further examined the incidence of CKD in non-CKD populations among patients over 50 years of age. We analyzed 1856 women and 1852 men participating in the epidemiology and risk factors surveillance of the CKD database. Women showed a significantly higher risk of renal progression and CKD than men within the hyperuricemia group. After adjusting covariates, women, but not men resulted in an hazard ratio (HR) for developing renal progression (HR = 1.12; 95% CI 1.01–1.24 in women and HR = 1.03; 95% CI 0.93–1.13 in men) and CKD (HR = 1.11; 95% CI 1.01–1.22 in women and HR = 0.95; 95% CI 0.85–1.05 in men) for each 1 mg/dL increase in serum urate levels. The association between serum urate levels and renal progression was stronger in women. Given the prevalence and impact of kidney disease, factors that impede optimal renal function management in women and men must be identified to provide tailored treatment recommendations.

## 1. Introduction

Uric acid is the final product of the breakdown of an exogenous pool of purines and endogenous purine metabolism [1], and most uric acid is excreted via the kidneys [2]. Elevated serum urate concentrations are associated with gout [3], diabetes mellitus [4,5], hypertension [6,7], metabolic syndrome [8] and cardiovascular mortality [9,10]. A meta-analysis of 1,134,073 participants showed a positive dose-response association between uric acid levels and cardiovascular disease mortality risk [11]. Hyperuricemia is common in chronic kidney disease (CKD) patients [12], and several studies have also shown a relationship between serum urate levels and kidney damage [13,14,15,16]. Urate-lowering therapy statistically significantly improved estimated glomerular filtration rate (eGFR) and serum creatinine and serum creatinine [17], and reduced the incidence of kidney failure events [18]. Uric acid crystallizations induce endothelial dysfunction [19,20], systemic inflammation [21], oxidative stress, and renin-angiotensin-aldosterone system activity [22], which may provide a pathogenic mechanism for chronic urate nephropathy.

Previous evidence has suggested that there are sex differences in uric acid levels; men have a higher uric acid level than women [23], and men have a higher prevalence of gout than women [24]. Sex differences decline with increasing age, but men still far exceed women in the prevalence of gout, even in older populations [24]. Age-related factors and hormone are also associated with uric acid levels [25]. Premenopausal women have lower levels of uric acid than men or postmenopausal women because estrogens stimulate kidney clearance, secretion and reabsorption [25,26]. However, there is evidence that postmenopausal women are at higher risk of gout than men [27]. Moreover, women with hyperuricemia had more comorbidities than men with hyperuricemia. Women with high uric acid levels had a higher risk of developing metabolic syndrome [28], diabetes [29,30], hypertension [7], coronary artery disease [31,32], and cardiovascular events [9,23] than men. Sex differences have appeared in the physiological regulation of urate homeostasis. Estrogen may play an important role in the regulation of the expression or activity of urate transporters [33].

In addition, the effects of uric acid on renal function are sex-specific. Both high and low uric acid levels increase the risk of developing chronic kidney disease (CKD) in men, while only high levels are a risk factor in women [34]. A historic cohort study in Japan indicated that uric acid levels greater than 6.0 mg/dL are associated with kidney impairment in men with mild kidney dysfunction [13]. However, a screened study conducted in Japan showed that higher uric acid levels increase the risk of developing ESRD in women but not in men [35].

Sex-specificity exists in renal progression in patients with early CKD [36,37]. In fact, whether the incidence of CKD and renal progression differ between postmenopausal women and men with hyperuricemia is unclear. Early detection of loss of kidney function and early treatment can decrease the risk of complications [38], prevent disease progression and prevent high medical costs in later stages [39]. Therefore, we investigated sex differences in the relationship between hyperuricemia and renal progression in early CKD and non-CKD populations, and further examined the incidence of CKD in non-CKD populations among patients over 50 years of age.

## 2. Materials and Methods

### 2.1. Study Population

**Figure 1:**
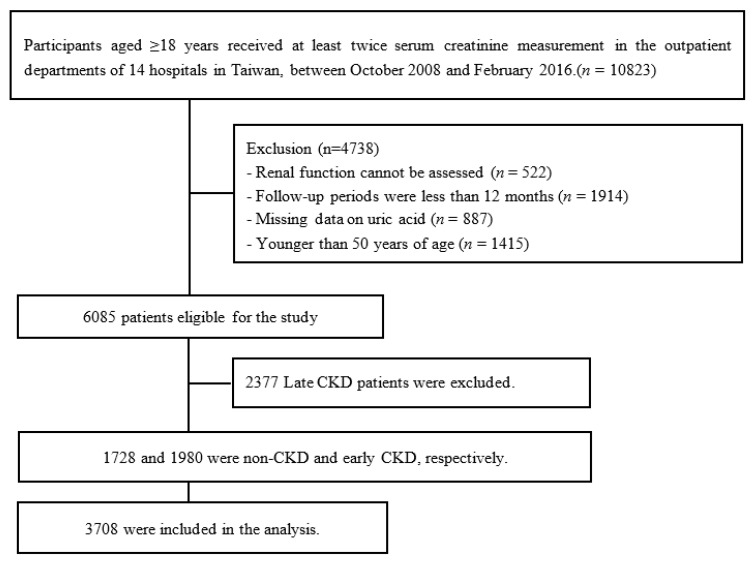
Flowchart of participants analyzed in this study.

A cohort based on the Epidemiology and Risk Factors Surveillance of CKD database was recruited from 14 medical centers and community in Taiwan, namely Tri-Service General Hospital, Taipei Medical University Hospital, Shuang Ho Hospital, Taipei Chang Gung Memorial Hospital of the C.G.M.F., Cardinal Tien Hospital, E-DA Hospital, National Cheng Kung University Hospital, China Medical University Hospital, Show Chwan Memorial Hospital, Changhua Christian Hospital, Taipei Veterans General Hospital, Kaohsiung Medical University Chung-Ho Memorial Hospital, Kaohsiung Municipal Siaogang Hospital, and Kaohsiung Chang Gung Memorial Hospital from October 2008 to February 2016. A total of 10,823 participants aged ≥18 years who had at least two serum creatinine measurements were recruited in this study. For this study, we excluded patients according to the following criteria: renal function could not be assessed, the follow-up periods were less than 12 months, missing data on uric acid or younger than 50 years of age. In total, 6085 patients were enrolled, with 1728, 1980 and 2377 having non-CKD, early CKD and late CKD, respectively. In the present study, we examined the risk factors for renal progression in subjects with non-CKD and early CKD, as well as the incidence of CKD in subjects with non-CKD (Figure 1).

The studied medical centers used the same medical laboratory criteria and protocols, and thus, the serum creatinine values from the different medical centers could be compared and standardized. We measured eGFR changes at the individual level and re-examined patients at the same medical center to control individual variation. After a thorough explanation of the study, all participants provided informed consent before data collection. This study was approved by the Ethics Committees of the Tri-Service General Hospital (TSGHIRB 100-05-197), Taipei Medical University (TMU-JIRB 20124036), Kaohsiung Medical University Chung-Ho Memorial Hospital (KMUHIRB 20120019), National Cheng Kung University Hospital (A-ER-101-117), Changhua Christian Hospital (CCHIRB 120405), Kaohsiung Chang Gung Memorial Hospital (101-1096B), Cardinal Tien Hospital (TMU-JIRB 201204035) and China Medical University Hospital (DMR101-IRB2-273(CR-1)). All the research methods were implemented in accordance with guidelines approved by the Ethics Committees.

### 2.2. Study Variables and Definitions

The definition of CKD was based on the Kidney Disease Outcomes Quality Initiative guidelines [40]. eGFR was calculated using the chronic nephrology epidemiology collaboration (CKD-EPI) equation [eGFR (mL/min/1.73 m^2^) = 141 × min (serum creatinine (SCr)/κ, 1)^α^ × max SCr/κ, 1)^−1.209^ × 0.993^age^ × 1.018 (if women) × 1.159 (if black)], κ = 0.7 (women) and 0.9 (men), α = −0.329 (women) and −0.411(men); min indicates the minimum of SCr/κ or 1, and max indicates the maximum of SCr/κ or 1 [41]. CKD stage 1 was defined as a eGFR value of ≥90 mL/min/1.73 m^2^ and the presence of kidney damage (i.e., proteinuria dipsticks ≥1+, urine protein-to-creatinine ratio (UPCR) ≥150, or urine albumin-to-creatinine ratio (UACR) ≥ 30); CKD stage 2 was defined as eGFR = 60–89 mL/min/1.73 m^2^ and the presence of kidney damage (i.e., proteinuria dipsticks ≥1+, urine protein-to-creatinine ratio (UPCR) ≥150, or urine albumin-to-creatinine ratio (UACR) ≥30); CKD stage 3a was defined as eGFR = 45–59 mL/min/1.73 m^2^; CKD stage 3b was defined as eGFR = 30–44 mL/min/1.73 m^2^; CKD stage 4 was defined as eGFR = 15–29 mL/min/1.73 m^2^; and CKD stage 5 was defined as eGFR <15 mL/min/1.73 m^2^ [42]. CKD stages 1, 2, and 3a referred to patients with early CKD, whereas CKD stages 3b, 4, and 5 referred to patients with late CKD.

According to the baseline eGFR, renal progression was defined as an eGFR that was reduced by more than 25% [43]. Non-CKD patients were followed up from the baseline measurement of renal function to the development of renal progression and CKD, or the end of the study period. Early CKD patients were followed up from the baseline measurement of renal function to the development of renal progression, or the end of the study period. The patients were censored at the end of the follow-up period.

Uric acid status was classified as the absence of hyperuricemia (serum urate concentration values of <7.0 mg/dL for men and <6.0 mg/dL for women) or the presence of hyperuricemia (serum urate concentration values of ≥7.0 mg/dL for men and ≥6.0 mg/dL for women) [44,45].

Sociodemographic characteristics, comorbidities, and health-related behaviors, including age, sex, hypertension, diabetes mellitus, dyslipidemia, CKD, gout, stroke, cigarette smoking, and alcohol consumption, were obtained at baseline using a structured questionnaire. Serum urate levels and physical examination variables, including height, weight, body mass index (BMI), serum creatinine, baseline eGFR, systolic blood pressure (SBP), diastolic blood pressure (DBP), fasting glucose, and total cholesterol, were obtained via medical chart review. Cigarette smoking was divided into never smoking, current smoker (had smoked at least 100 cigarettes in the lifetime and is currently smoking cigarettes) or former smoker (had smoked at least 100 cigarettes in the lifetime but had quit smoking at the time of the interview) [46,47]. Alcohol consumption was divided into current alcohol consumers versus participants who never consumed alcohol.

### 2.3. Statistical Analysis

To determine the significant differences between women and men, participants were examined using the chi-square test and Student’s t test for categorical and continuous variables, respectively. The incidence density method was used to estimate the uric acid status and sex-specific incidence rates of renal progression and CKD. Multivariate Cox proportional hazards analysis was used to estimate the hazard ratio (HR) and 95% confidence interval (CI) of the risk of renal progression and CKD associated with sex. Covariates included age, CKD, hypertension, diabetes mellitus, dyslipidemia, gout, stroke, BMI, serum creatinine, cigarette smoking, and alcohol consumption.

To induce a similar distribution of covariates between women and men, we used the inverse probability of treatment weighting (IPTW) analysis method, where the weight was the inverse (i.e., reciprocal) of the propensity scores [48]. To assess the balance of baseline covariates between women and men, we applied absolute standardized mean differences. In addition, the value of the absolute standardized mean difference was required to be lower than 10% between the sexes, indicating negligible differences among the covariates. In addition, the value of the absolute standardized mean difference was less than 10% between sexes, indicating negligible differences among the potential confounders [49]. The risks of renal progression and CKD among the groups of women and men were compared using Cox proportional hazards regression with IPTW. In order to compare the results of premenopausal and postmenopausal women, we further analyzed the sex differences in the relationship between hyperuricemia and renal progression and the incidence of CKD among patients before 50 years of age. Analyses were performed using SAS software (SAS Institute), version 9.4.

## 3. Results

**Figure 2:**
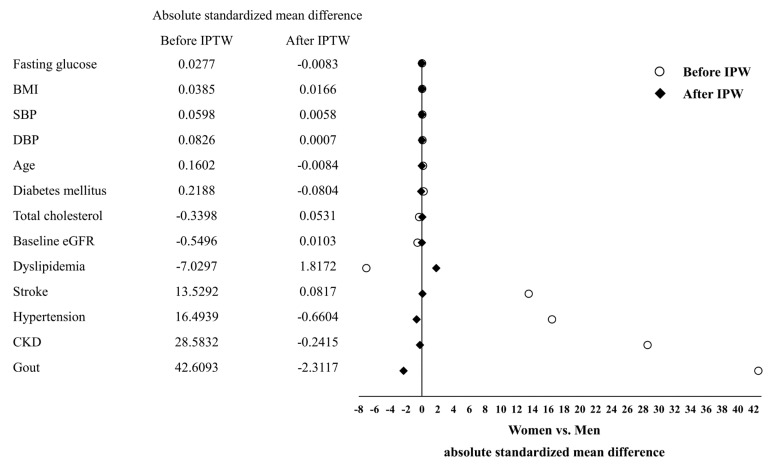
Women and men by characteristics before and after propensity weighting.

**Figure 3:**
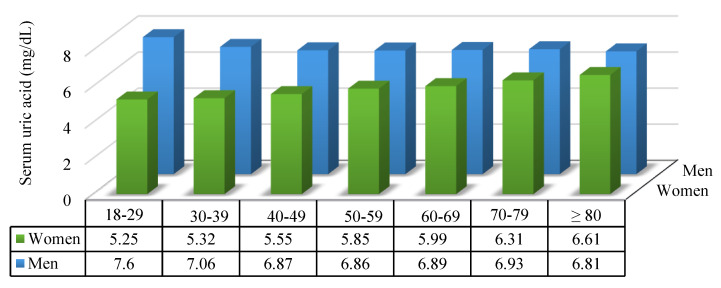
Bar chart of uric acid concentrations in each age group.

Table 1 shows the demographic and clinical characteristics of the women and men. Of the 3708 participants, 1856 (50.05%) and 1852 (49.95%) were women and men, respectively. Before employing IPTW, the women exhibited significantly lower prevalences of hypertension (1124 (60.56%)), CKD (859 (46.28%)), gout (118 (6.36%)), stroke (87 (4.69%)), current smoking (36 (1.96%)), and alcohol consumption (63 (3.43%)) as well as low levels of serum urate (5.49 (SD 1.36)), serum creatinine (0.73 (SD 0.15)), and DBP (76.02 (SD 10.71)) than men (Table 1). After applying IPTW, the different sexes were well balanced with respect to key characteristics, such as fasting glucose, BMI, SBP, DBP, age, diabetes mellitus, total cholesterol, baseline eGFR, dyslipidemia, stroke, hypertension, CKD, and gout (all absolute standardized mean difference lower than 10%; Figure 2). The mean levels of uric acid in men was higher than that in women in each age group (18–29 group, *p* < 0.001; 30–39 group, *p* < 0.001; 40–49 group, *p* < 0.001; 50–59 group, *p* < 0.001; 60–69 group, *p* < 0.001; 70–79 group, *p* < 0.001; ≥80, *p* = 0.214), but levels of uric acid were increases with advancing age in women but not in men. Both in women and men, uric acid levels showed statistically significant differences in each age group (women, *p* < 0.001; men *p* = 0.004) (Figure 3).

Within the group without hyperuricemia, the incidence rates of renal progression in men and women were 4.86 and 3.79 per 100 patient-years, respectively; the incidence rates of CKD in men and women were 13.31 and 9.54 per 100 patient-years, respectively. Within the hyperuricemia group, the incidence rates for renal progression in men and women were 5.44 and 6.15 per 100 patient-years, respectively; the incidence rates for CKD in men and women were 14.54 and 15.26 per 100 patient-years, respectively (Table 2).

After using IPTW, women showed a significantly higher risk of renal progression (HR 2.21; 95% CI 1.55–3.14) and CKD (HR 2.69; 95% CI 1.90–3.81) than men within the group without hyperuricemia. Women showed a significantly higher risk of renal progression (HR 2.14; 95% CI 1.35–3.40) and CKD (HR 3.56; 95% CI 2.04–6.25) than men within the hyperuricemia group (Table 3).

**Figure 4:**
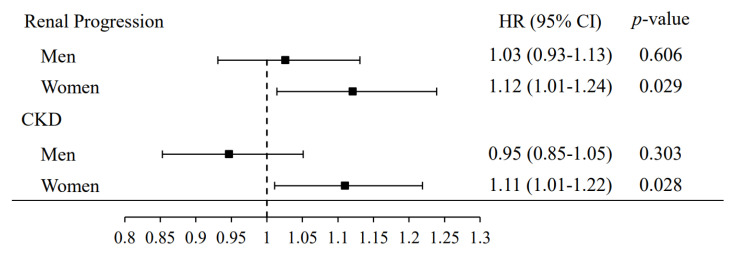
Risk of renal progression and CKD per 1 mg/dL increase in serum uric acid by sex. Adjusted for age, CKD, hypertension, diabetes mellitus, dyslipidemia, gout, stroke, BMI, serum creatinine, cigarette smoking, and alcohol consumption.

In women, the multivariate adjustment for age, CKD, hypertension, diabetes mellitus, dyslipidemia, gout, stroke, BMI, serum creatinine, cigarette smoking, and alcohol consumption resulted in an HR for developing renal progression of 1.12 (95% CI, 1.01–1.24) and for CKD of 1.11 (95% CI, 1.01–1.22) for each 1 mg/dL increase in serum urate levels. In men, there was no association between serum urate levels and developing renal progression and CKD (Figure 4).

We further analyzed the results of patients before the age of 50. Women did not have a higher risk of renal progression and CKD than men within the group without hyperuricemia (Table S1).

## 4. Discussion

The multicenter cohort study enrolled subjects with early CKD and without CKD to demonstrate that serum urate levels are an independent predictor of renal progression and incident CKD. We found significant differences between men and women. The effect of sex differences in the role of serum urate levels on the long-term prognosis of patients in terms of renal progression and incident CKD among early CKD and non-CKD has not previously been fully explored in middle-aged and elderly adults. To our knowledge, this is the first study to elucidate sex differences in serum urate levels for the prognosis of patients with kidney damage among middle-aged and elderly adults with early CKD and without CKD. 

Serum urate levels are a well-known marker of hypertension, gout, arterial stiffness, cardiac hypertrophy, metabolic syndrome, and cardiovascular damage [3,8,9,50,51,52,53]. Our findings on the effect of serum urate levels on the risk of renal progression among individuals with early CKD are consistent with previous studies. In patients with renal disease, a higher uric acid concentration was a strong independent predictor of kidney failure in earlier stages of CKD [16]. Srivastava et al. described this relationship of uric acid concentration with all-cause mortality as J-shaped in individuals with CKD [16]. High uric acid was found to be a risk factor for CKD and a rapid decline in eGFR in patients with mild kidney dysfunction [13]. We observed that hyperuricemia is an independent risk factor for incident CKD among individuals without CKD. In the general population, uric acid levels promote initial kidney damage and incident kidney disease [14,54,55].

However, a recently meta-analysis of randomized controlled trials demonstrated that urate-lowering therapy was associated with slowing the decline rate of GFR, but there is insufficient evidence to support urate lowering in patients to improve kidney outcomes [12]. Previous Mendelian randomized study found that there is no evidence to support a causal relationship between serum uric acid levels and eGFR levels or incident CKD [56]. The Controlled Trial of Slowing of Kidney Disease Progression from the Inhibition of Xanthine Oxidase (CKD-FIX) demonstrated that allopurinol dose not slow down the progression of CKD in patients with severe CKD and high risk of progression [57]. The Preventing Early Renal Loss in Diabetes (PERL) trial did not show a beneficial effect of reduction of the serum urate level with allopurinol therapy on the kidney outcomes in persons with type 1 diabetes and early-to-moderate diabetic kidney disease [58]. The PERL and CKD-FIX trials do not support the clinical benefits of allopurinol in the treatment of asymptomatic hyperuricemia on kidney outcomes [57,58].

Although, the causal relationship between the effects of uric acid and CKD is still debatable. In a perspectives article argues that hyperuricemia is directly related to the development and progression of CKD, and treatment with urate-lowering therapy may provide clinical benefits [59]. Hyperuricemia has a detrimental effect on kidney function [59].

Hyperuricemia accelerates renal progression by increasing renal renin and cyclooxygenase-2 expression and causing vascular smooth muscle cell proliferation [60]. Oxidative stress plays an important pathophysiological role in renal progression [22]. Furthermore, impaired kidney clearance is responsible for increased uric acid concentrations [16].

There are sex differences in hypertension and cardiovascular events in patients with high serum urate levels, and the rates in female patients are significantly higher than those in males [9,61]. The present study demonstrated that sex differences in the association between uric acid level and renal progression and incident CKD were stronger in women than in men. A previous study in Okinawa, Japan also found a significant association between uric acid and renal failure requiring renal replacement in women, but no significant association was observed in men [35].

It is well known that men have higher uric acid levels than women. Our findings also showed that men had higher levels of uric acid than women in each age group; however, we found that uric acid levels increased with advancing age in women but not in men. Women before the age of 50 did not have a higher risk of renal progression and CKD than men. Uric acid levels increase with age, and after menopause, the levels roughly equal the levels in men in later years [62]. A previous study in Japan showed that women uric acid levels increased until the age of 70 years or older as a result of changes in hormones accompanying menopause [63]. This finding provides a potential explanation for why women have a higher risk of kidney damage than men.

Young women have higher levels of estrogen, which can promote renal uric acid excretion and reduce uric acid levels [26,64]. The Third National Health and Nutrition Examination Survey reported that postmenopausal women receiving hormone replacement therapy may have reduced uric acid levels [25]. Further investigations are encouraged to investigate the mechanism by which hyperuricemic women have a higher risk of reduced renal function than men among elderly populations.

Our findings indicate that young men aged 18–29 and 30–39 have higher serum urate levels and decrease with age, while the opposite is the case in women. If young people aged 18–40 have normal kidney function and they develop hyperuricemia, the most likely clinical indication is genetic variants in urate transporters, such as SLC2A9 [65], rather than underlying chronic kidney disease. Additionally, there is extensive evidence that variants in ABCG2 cause hyperuricemia in young people [66,67,68]. Thus, predicting whether these patients develop CKD or whether hyperuricemia contributes to CKD progression later in life is impossible due to genetic components and environmental factors as previously described in a study in Taiwan [69]. Therefore, in this study, the sex-specific relationship between hyperuricemia and renal progression was studied, with a focus on patients over 50 years of age.

In addition, we found that younger women with hyperuricemia have a higher risk of kidney deterioration than men with hyperuricemia. A study of a military cohort of young individuals also found that the association between hyperuricemia and raised blood pressure was stronger in women than in men [70]. One possible explanation for the sex difference is that women with hyperuricemia may have higher levels of xanthine oxidase activity and reactive oxygen species productions than men, leading to more severe renal vascular inflammation in women than in men [71,72].

This study has several advantages. First, we had a large cohort that recruited patients from multiple centers throughout Taiwan. Second, we provided sex-specific data showing a difference in the relationship between hyperuricemia and renal progression. Third, this study used well-trained interviewers to collect data on demographic characteristics and health-related behaviors face to face. Finally, in order to reduce the effects of confounding in observational study, we used the IPTW analysis method to induce similar covariate distribution between women and men.

However, our study has some limitations. First, this research may have potential selection bias because our patients were voluntarily recruited. Second, certain test results may be underestimated due to the use of structured questionnaires to collect clinical disease data. Third, this study does not contain information on potential factors, including menopausal age and hormone replacement therapy. Fourth, this study uses structured questionnaires to collect alcohol consumption data, but does not include daily alcohol consumption. Therefore, it is difficult to accurately analyze the possible relationship between alcohol consumption with hyperuricemia and renal decline. Therefore, these factors should be considered in future research.

## 5. Conclusions

Given the prevalence and impact of kidney disease, factors that impede optimal renal function management in women and men must be identified to provide tailored treatment recommendations. Elevated serum urate levels are a strong risk factor for developing kidney disease. Therefore, our results suggest that individuals, especially postmenopausal women, with higher uric acid levels are at increased risk of developing renal progression and incident CKD.

## Figures and Tables

**Table 1:** The baseline characteristics subjects by sex.

Characteristic	Women	Men	*p*-Value
	(*n* = 1856)	(*n* = 1852)	
Serum uric acid (mg/dL)	5.49 ± 1.36	6.36 ± 1.39	<0.001 ***
Uric Acid status, %			0.369
Without hyperuricemia	1235 (66.54)	1259 (67.98)	
With hyperuricemia ^a^	621 (33.46)	593 (32.02)	
Follow-up duration (month)	33.17 (15.30)	34.18 (15.42)	0.046 *
Age (years)	63.77 ± 8.57	65.21 ± 9.41	<0.001 ***
Comorbidities, %			
Hypertension	1124 (60.56)	1270 (68.57)	<0.001 ***
Diabetes mellitus	766 (41.27)	768 (41.47)	0.930
Dyslipidemia	672 (36.21)	610 (32.94)	0.040 *
Early-CKD	859 (46.28)	1121 (60.53)	<0.001 ***
Gout	118 (6.36)	382 (20.63)	<0.001 ***
Stroke	87 (4.69)	148 (7.99)	<0.001 ***
Physical examination			
Height (cm)	154.92 ± 5.66	165.89 ± 6.29	<0.001 ***
Weight (kg)	60.69 ± 10.65	70.03 ± 11.04	<0.001 ***
BMI (kg/m^2^)	25.27 ± 4.20	25.41 ± 3.50	0.276
Serum creatinine (mg/dl)	0.73 ± 0.15	1.02 ± 0.19	<0.001 ***
Baseline eGFR (mL/min per 1.73 m^2^)	73.63 ± 12.07	67.05 ± 11.87	<0.001 ***
SBP (mmHg)	130.17 ± 18.16	131.21 ± 16.34	0.092
DBP (mmHg)	76.02 ± 10.71	76.92 ± 11.07	0.020 **
Fasting glucose (mg/dl)	113.91 ± 37.25	114.91 ± 34.56	0.410
Total cholesterol (mg/dL)	192.45 ± 39.20	179.27 ± 38.37	<0.001 ***
Health-related behaviors, %			
Cigarette smoking			<0.001 ***
Never smoker	1801 (97.88)	1153 (62.94)	
Current smoker	36 (1.96)	451 (24.62)	
Former smoker	3 (0.16)	228 (12.62)	
Alcohol consumption	63 (3.43)	353 (19.34)	<0.001 ***

Abbreviations: CKD, chronic kidney disease; BMI, body mass index; SBP, systolic blood pressure; DBP, diastolic blood pressure. ^a^ Female ≥ 6.0 mg/dL; Male ≥ 7.0 mg/dL. * *p* < 0.05, ** *p* < 0.01, *** *p* < 0.001.

**Table 2:** Incidence rate for risk of renal progression (25% decline in GFR) and CKD by uric acid in women and men.

Gender	Renal Progression (*n* = 3708)			CKD (*n* = 1728)		
	Events, *n*(%)	Person-Years	IR (95% CI) ^a^	Events, *n*(%)	Person-Years	IR (95% CI) ^a^
Without Hyperuricemia	305			373		
Men	176 (57.70)	3619.9	4.86 (4.17–5.64)	189 (50.67)	1420.5	13.31 (11.48–15.34)
Women	129 (42.30)	3405.6	3.79 (3.16–4.50)	184 (49.33)	1927.9	9.54 (8.22–11.03)
With Hyperuricemia ^b^	196			164		
Men	90 (45.92)	1655.5	5.44 (4.37–6.68)	69 (42.07)	474.4	14.54 (11.32–18.41)
Women	106 (54.08)	1724.9	6.15 (5.03–7.43)	95 (57.93)	622.6	15.26 (12.35–18.65)

Abbreviation: CKD, chronic kidney disease; CI, confidence interval; IR, incidence rate. ^a^ IR/100 person-years. ^b^ Female ≥ 6.0 mg/dL; Male ≥ 7.0 mg/dL.

**Table 3:** Risk of renal progression and CKD by hyperuricemia in women and men.

Gender	Renal Progression, HR (95% CI)				CKD, HR (95% CI)			
	Before Propensity Weighting		After Propensity Weighting		Before Propensity Weighting		After Propensity Weighting	
	Crude	Adjusted ^a^	Crude	Adjusted ^a^	Crude	Adjusted ^b^	Crude	Adjusted ^b^
Without Hyperuricemia								
Men	1.00	1.00	1.00	1.00	1.00	1.00	1.00	1.00
Women	0.81 (0.65–1.02)	1.93 (1.35–2.77) ^e^	1.42 (1.10–1.82) ^d^	2.21 (1.55–3.14) ^e^	0.71 (0.58–0.87) ^e^	2.35 (1.63–3.40) ^e^	1.01 (0.81–1.26)	2.69 (1.90–3.81) ^e^
With Hyperuricemia								
Men	1.00	1.00	1.00	1.00	1.00	1.00	1.00	1.00
Women	1.14 (0.86–1.51)	2.14 (1.30–3.52) ^d^	1.28 (0.95–1.73)	2.14 (1.35–3.40) ^d^	1.10 (0.80–1.50)	4.05 (2.17–7.56) ^e^	1.42 (1.00–2.00) ^c^	3.56 (2.04–6.25) ^e^

Abbreviation: CKD, chronic kidney disease; HR, hazard ratio; CI, confidence interval. ^a^ Adjusted for age, CKD, hypertension, diabetes mellitus, dyslipidemia, gout, stroke, BMI, serum creatinine, cigarette smoking, and alcohol consumption. ^b^ Adjusted for age, hypertension, diabetes mellitus, dyslipidemia, gout, stroke, BMI, serum creatinine, cigarette smoking, and alcohol consumption. ^c^ *p* < 0.05; ^d^ *p* < 0.01; ^e^ *p* < 0.001.

## Data Availability

The data are not publicly available due to subject’s privacy.

## Supplementary Materials

The following are available online at https://www.mdpi.com/article/10.3390/jpm11050415/s1, Table S1: Risk of renal progression and CKD by hyperuricemia in younger women and men (Less than 50 years old).
