# Supplementary material for: Sex-Specific Association of Uric Acid and Kidney Function Decline in Taiwan
Source: J Pers Med. 2021 May 15;11(5):415. doi: 10.3390/jpm11050415 (PMC8156506; doi:10.3390/jpm11050415)
Supplement: Supplementary file 1 [file jpm-11-00415-s001.zip › jpm-1187318-supplementary.pdf]

Table S1. Risk of renal progression and CKD by hyperuricemia in younger women and men (Less than 50 years old).

Gender	Renal Progression, HR (95% CI)				CKD, HR (95% CI)			
	Before propensity weighting		After propensity weighting		Before propensity weighting		After propensity weighting	
	Crude	Adjusted [a]	Crude	Adjusted [a]	Crude	Adjusted [b]	Crude	Adjusted [b]
Without Hyperuricemia								
Men	1.00	1.00	1.00	1.00	1.00	1.00	1.00	1.00
Women	0.56 (0.30-1.02)	1.95 (0.74-5.19)	1.15 (0.55-2.40)	1.88 (0.68-5.23)	0.53 (0.32-0.90)	1.60 (0.57-4.51)	0.86 (0.50-1.48)	2.11 (0.85-5.22)
With Hyperuricemia								
Men	1.00	1.00	1.00	1.00	1.00	1.00	1.00	1.00
Women	0.96 (0.52-1.78)	1.51 (0.65-3.54)	1.30 (0.71-2.39)	2.58 (1.18-5.64)	0.67 (0.29-1.53)	2.51 (0.57-11.03)	1.09 (0.49-2.44)	5.49 (1.11-27.19)

Abbreviation: CKD, chronic kidney disease; HR, hazard ratio; CI, confidence interval

[a] Adjusted for age, CKD, hypertension, diabetes mellitus, dyslipidemia, gout, stroke, BMI, serum creatinine, cigarette smoking, and alcohol consumption.

[b] Adjusted for age, hypertension, diabetes mellitus, dyslipidemia, gout, stroke, BMI, serum creatinine, cigarette smoking, and alcohol consumption.
